# Effect of Hollow Corundum Microspheres Additive on Physical and Mechanical Properties and Thermal Shock Resistance Behavior of Bauxite Based Refractory Castable

**DOI:** 10.3390/ma14164736

**Published:** 2021-08-22

**Authors:** Rimvydas Stonys, Jurgita Malaiškienė, Jelena Škamat, Valentin Antonovič

**Affiliations:** Institute of Building Materials, Vilnius Gediminas Technical University, 08217 Vilnius, Lithuania; rimvydas.stonys@vilniustech.lt (R.S.); jelena.skamat@vgtu.lt (J.Š.); valentin.antonovic@vgtu.lt (V.A.)

**Keywords:** refractory castable, hollow corundum microspheres, bauxite aggregate, thermal shock resistance

## Abstract

This paper analyses the effect of hollow corundum microspheres (HCM) on both physical-mechanical properties (density, ultrasonic pulse velocity, modulus of elasticity, and compressive strength) and thermal shock resistance behavior of refractory medium cement castable with bauxite aggregate. Moreover, the scanning electron microscopy (SEM) results of HCM and refractory castable samples are presented in the paper. It was found that the replacement of bauxite of 0–0.1 mm fraction by HCM (2.5%, 5%, and 10% by weight of dry mix) had no significant effect on the density and compressive strength of castable, while the modulus of elasticity decreased by 15%. Ultrasonic pulse velocity (*Vup*) values and the visual analysis of the samples after thermal cycling showed that a small amount of HCM in composition of refractory castable could reduce the formation and propagation of cracks and thus increase its thermal shock resistance.

## 1. Introduction

Most high temperature processes in industrial furnaces are combined with cycles of heating and cooling. In these applications, refractory materials are exposed to temperature gradient during operation, which causes thermal stresses and damage to the material. Therefore, the durability of the lining significantly depends on the thermal shock resistance of refractory materials used in thermal units. The thermal shock resistance of castable depends on many factors: chemical composition, microstructure, phase transformation at high temperature during firing process, etc. [1,2]. 

The authors [3] point out that about one-third of refractories fail due to insufficient thermal shock resistance. The destruction of refractory material is regarded as a two-stage process that includes crack formation, its further growth and propagation. Based on the thermoelastic theory of crack nucleation, the stress-to-elastic modulus ratio (σ/E) is used to assess the ability of a material to resist crack nucleation [4,5]. This means that a stronger material with a lower modulus of elasticity will have a higher thermal resistance. At the same time, the characteristics of the material’s microstructure make it possible to control the propagation of cracks [6,7,8]. Thus, the formation of a heterogeneous structure by combining components with rather different coefficients of thermal expansion makes it possible to obtain a system of microstructurally small cracks that reduce the modulus of elasticity and effectively inhibit the propagation of larger cracks [5,9]. It has been noted that thermal shock resistance of a material can be improved by creating a fragmentary structure [10] and by reinforcing it with fibers of different nature: metallic, ceramic, and carbon [11,12]. A structure of controlled fragmentation is more mobile and the grains and crystals in such a structure can expand freely without causing additional stresses. As reinforcing inclusions often have a higher strength than the refractory matrix, they not only inhibit crack propagation in the material, but also inhibit the composite’s failure. 

The presence of pores not only improve the thermal resistance of the material but also reduce stress levels and slow down the propagation of micro-cracks [13,14]. This positive role of pores is achieved if they are round and of quite a big size. Pores of a regular spherical shape can significantly reduce the level of stress concentration, as the propagation of the crack that enters the pore is stopped or suspended. 

In terms of increasing the thermal shock resistance of refractories by inhibiting cracks, a certain positive effect can be achieved by using additives with particle of a spherical shape, such as hollow corundum microspheres (HCM), which, also, have a low thermal conductivity and low sintering activity [15,16]. Authors of [17] found that HCM improve mullite castables’ thermal shock resistance and explain this effect by the strong bonding at the microsphere and matrix interface, when the crack propagation is stopped at the corundum microsphere. It is also found that the addition of HCM has a positive effect on the strength of various types of materials. Authors of [18] found that the addition of microporous corundum contributes to increase the strength of refractory castable with spinel. The study of corundum modified refractories [19] revealed that the smaller the size of corundum spheres (spheres ranging in size from 100 to 2000 microns were studied) the stronger their effect on increasing the strength of refractory. 

Thermal shock resistance of refractories is determined using tests in which the material is heated and cooled, and the number of cycles that a material can withstand prior to failure and without spalling, is taken as its resistance to thermal shock. However, experimental method of thermal shock resistance evaluation is both expensive and time-consuming [20]. The level of degradation of the samples can also be monitored before and during testing by nondestructive test, such as ultrasonic pulse velocity technique [21]. The formation of the cracks has a significant impact on the ultrasonic velocity and the Young’s modulus of the refractories. The revealed correlation between thermomechanical properties, *Vup*, microstructure, and the nature of crack propagation makes it possible to predict the thermal shock resistance of the material without performing a large number of experimental cycles.

In the experiments described in this paper, the addition of corundum microspheres was used to improve both the physical and mechanical properties as well as the thermal resistance of medium cement castable with bauxite aggregate.

## 2. Materials and Methods

The following materials were used to make the samples.

High alumina cement Gorkal-70 (G70) (chemical composition, mass %: Al_2_O_3_—71.0; CaO—28.0; SiO_2_—0.5, and Fe_2_O_3_—0.4. Blaine surface area 450 m^2^/kg, bulk density 1100 kg/m^3^) manufactured by Górka Cement Sp. zo.o. (Trzebinia, Poland).

Microsilica (MC) RW-Fuller (chemical composition, mass %: SiO_2_—96.1, Al_2_O_3_—0.2, Fe_2_O_3_—0.1, C—0.6, CaO—0.3, MgO—0.4, K_2_O—1.2, Na_2_O—0.1, and SO_3_—0.3) manufactured by RW Silicium GmbH (Pocking, Germany).

Reactive alumina (RA) CTC 20 (chemical composition, mass %: Al_2_O_3_—99.7; Na_2_O—0.1; Fe_2_O_3_—0.03; SiO_2_—0.03; and CaO—0.02. Blaine surface area 2100 m^2^/kg) manufactured by Almatis (Ludwigshafen, Germany).

Calcined alumina (CA) CT 19 (chemical composition, mass %: Al_2_O_3_—99.8; Na_2_O—0.1; Fe_2_O_3_—0.02; SiO_2_—0.01; and CaO—0.03. Blaine surface area 400 m^2^/kg) manufactured by Almatis (Ludwigshafen, Germany).

Bauxite (chemical composition, mass %: Al_2_O_3_—81.7; SiO_2_—10.0; TiO_2_—4.4; Fe_2_O_3_—2.4; CaO—0.5; P_2_O_5_—0.3; K_2_O—0.3; MgO—0.2; ZrO_2_—0.2; Na_2_O—0.04; and SO_3_—0.02) of different fractions was used as a filler by Stanchem (Niemce, Poland).

**Figure 1:**
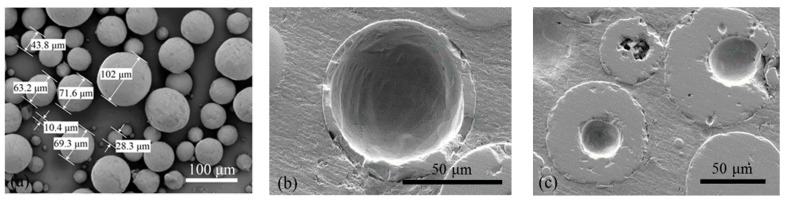
External (**a**) and internal (**b**,**c**) morphology of HCM (SEM images).

The mixtures were prepared using the following chemical additives: polycarboxylate ether (PCE) Castament FS 20 manufactured by BASF Construction Solutions GmbH (Trotsberg, Germany) and sodium tripolyphosphate (TP) Na_5_P_3_O_10_. Bauxite of different fractions was used as a filler. In experimental compositions 25%, 50%, and 100% of bauxite of 0–0.1 mm fraction (bulk density 1770 kg/m^3^) were replaced with hollow corundum microspheres (Kit-Stroi SPb, Saint Petersburg, Russia) with a particle size ranging from 5 to 100 µm (Figure 1a), which corresponds to 2.5%, 5%, and 10% of the dry mixture mass. The bulk density of the HCM is 1750 kg/m^3^.

SEM studies revealed that some of the HCM used in this work were solid. The hollow spheres had the wall thickness varying over a wide range (Figure 1b,c).

**Figure 2:**
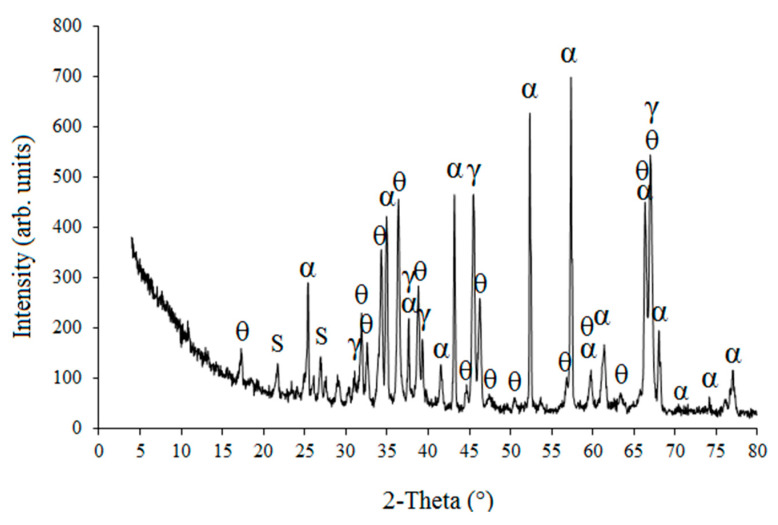
XRD pattern of HCM diffraction curve: α—α-Al_2_O_3_; γ—γ-Al_2_O_3_; θ—θ-Al_2_O_3_; S-SiO_2_.

According to the results of chemical analysis (by X-ray fluorescence spectroscopy (XRF)) HCM consists of 99.0 % of Al_2_O_3_; the rest are SiO_2_, Na_2_O, and ZrO_2_ (~0.65% in total) and such compounds as MgO, P_2_O_3_, SO_3_, K_2_O, CaO, Fe_2_O_3_, ZnO, Ga_2_O_3_, and Y_2_O_3_ (~0.35% in total). X-ray diffraction analysis (XRD) showed that the main phase of HCM is α-Al_2_O_3_ of trigonal crystal structure, which is a stable form of aluminum oxide. The diffraction curve also shows reflections that can be attributed to cubic (γ-Al_2_O_3_) and monocline (θ-Al_2_O_3_) modifications of aluminum oxide and reflections typical of SiO_2_ (Figure 2).

The castable mixes were prepared using potable water. Compositions of castables are given in Table 1. 

Castable dry materials were mixed for 5 min, then water was added and the mixing continued for another 5 min. Then, 70 mm × 70 mm × 70 mm samples were formed from the prepared mixture and kept in the mold for 24 h. Afterwards the samples were taken out, they were conditioned for 2 days at 20 ± 1 °C and dried at 110 ± 5 °C.

Samples (3 of each composition) for the tests of physical and mechanical properties according to LST EN ISO 1927-6:2013 were fired at 1100 ± 5 °C and 1300 ± 5 °C. The compressive strength was determined using the press ALPHA3-3000S (Riedlingen, Germany). 

The samples for thermal shock resistance tests were fired at 950 °C. Thermal shock resistance of castable was established for 3 samples of each composition in accordance with DIN 51068, according to which the samples were kept for 15 min at 950 °C and then cooled for 3 min in water (cycle 1). 

The destruction of the material was estimated by means of ultrasonic tests with the samples subjected to thermal cycling (3 of each composition). The ultrasonic pulse velocity (Vup, m/s) was calculated according to literature [22]. 

The compressive strength degradation was calculated according to Equation (1):

(1) $$\sigma =\sigma_{0}\cdot {\left(\frac{{\mathrm{Vup}}_{0}}{{\mathrm{Vup}}_{30}}\right)}^{n}$$

where: 𝜎_0_ is the compressive strength of the sample before exposure of the material to the thermal shock testing, MPa; Vup_0_ is the longitudinal velocity before testing, m/s; Vup_30_ is the longitudinal velocity after testing (30 cycles), m/s; *n* is the material constant (0.488) [23,24]. 

The modulus of elasticity was calculated from the equation [25] for 3 samples of each composition:

(2) $$E={\mathrm{Vup}}^{2}\cdot \rho \frac{\left(1+\mu \right)\left(1-2\mu \right)}{1-\mu }$$

where: Vup is ultrasonic pulse velocity, m/s; *ρ* is density, kg/m^3^; and µ is Poisson’s ratio of 0.17 for all castables.

Also, for the evaluation of material thermal shock resistance, the 𝜎/E ratio was calculated. 

Microstructure analysis of the materials was done with a scanning electron microscope SEM JEOL JSM-7600F (Tokyo, Japan) on splitting surfaces pre-coated with a conductive gold layer (QUORUM Q150R ES vacuum sputtering coating machine, Quorum technologies, Laughton, UK).

## 3. Results and Discussion

The replacement of the bauxite part by having the same bulk density HCM was not found to have a significant effect on the density of castables after hardening at 20 °C (~2730 kg/m^3^), drying at 110 °C (~2650 kg/m^3^), and after firing at different temperatures (at 1100 °C—~2590 kg/m^3^ and at 1300 °C—~2620 kg/m^3^). The difference in the average density values did not exceed 1%. 

**Figure 3:**
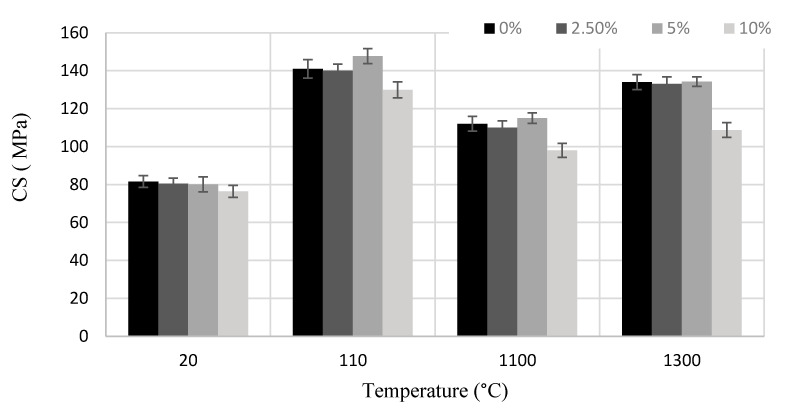
Dependence of compressive strength (CS) of the castable on the amount of HCM and temperature.

The mechanical testing of castable specimens showed (Figure 3) that up to 5% addition of HCM had no significant effect on the strength of the castable. A slight increase in compressive strength (CS) at 5% HCM after drying and firing at 1100 °C can be noted, but the difference with the control sample does not exceed 5%. In specimens containing up to 10% of HCM, a more expressed tendency of castable strength reduction was observed both after hardening and drying, as well as after firing at 1100 and 1300 °C. This reduction can be related to a clustering of HCM particles and formation of more voids in the sample matrix. 

**Figure 4:**
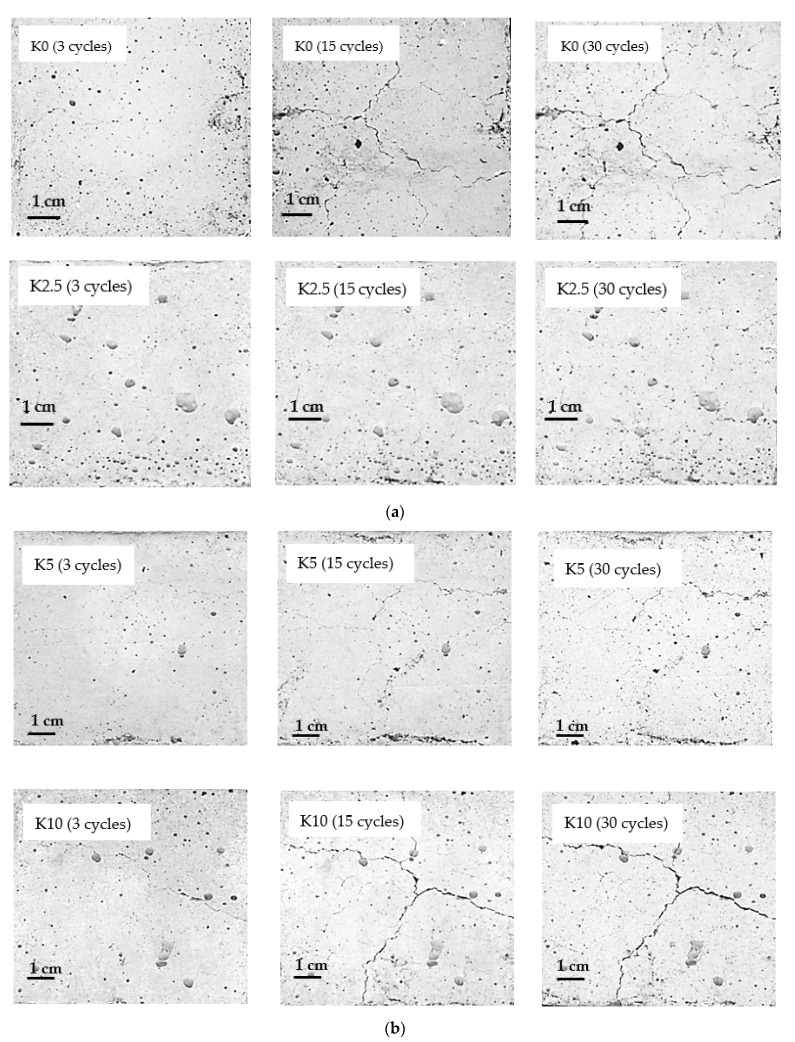
(**a**) Surfaces of castable specimens K0 and K2.5 after 3, 15, and 30 thermal cycles. (**b**) Surfaces of castable specimens K5 and K10 after 3, 15, and 30 thermal cycles.

The castable compositions investigated in this work withstood 30 cycles without significant signs of fracture. However, the formation of micro and macro cracks on the surface of the specimens was observed during thermal cycling and the length and width of the cracks increased with the number of thermal shocks. The difference in the damage of the different samples is well noticeable after 15 cycles and after 30 cycles the pattern of crack formation did not change much. The largest number of macro-cracks (Figure 4) was detected on the surfaces of control specimens (K0), as well as on specimens K10 containing the largest amount of HCM (10 wt. % of dry mix). The specimens with a lower HCM content of 2.5% and 5% (K2.5 and K5) were damaged the least. 

Both the material structural continuity and density determine ultrasonic wave velocity in materials: the structural damages disturb propagation of ultrasonic wave and decrease its velocity. The ultrasonic wave in material with defects reflect, refract, and diffract, which will lead to changes (decreases) in the propagation velocity of the ultrasonic waves [26,27]. 

**Figure 5:**
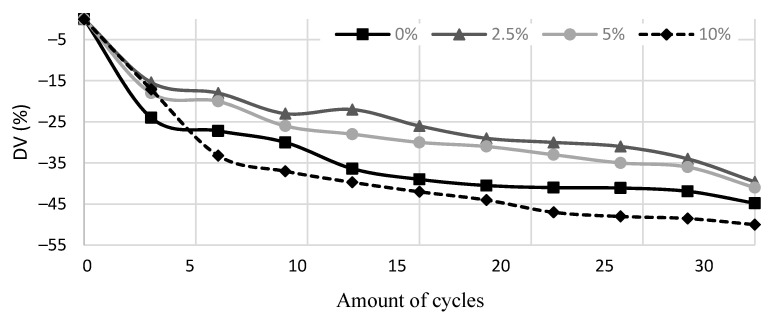
Variation of ultrasonic pulse velocity (DV) during thermal cycling of specimens with different HCM content.

The drop in Vup after firing at high temperatures is typical for refractory concretes: the discontinuity of microstructure increases due to destroying hydraulic bonds and evaporation of chemically combined hydraulic phases. The curves of ultrasonic pulse velocity variation DV (%) in the tested castable samples during thermal cycling are shown in Figure 5. The bigger decrease in ultrasonic pulse velocity corresponds to a bigger amount of microcracks and other discontinuities developed in material during thermal cycling and, respectively, lower material capability to withstand thermal shocks.

A sharp decrease of Vup was observed in all specimens in the first stage of thermal cycling. This decrease is associated with the formation of the first microcracks in the material. Vup continued decreasing slowly and gradually with the increasing number of thermal cycles during further testing caused by gradual degradation of castable structure and accumulation of micro and macro cracks. The most significant decrease of Vup (~50%) after 30 cycles was observed for specimens K10, which cracked the most compared to the rest of the specimens (Figure 4). In specimens K2.5 and K5, the surface of which showed less prominent formation of macrocracks after 30 thermal cycles, Vup reduced by ~40%. The control specimens showed an intermediate value of Vup decrease, ~45%. Applying Equation (1), the strength degradation after 30 cycles for K0, K2.5, K5, and K10 reach 83.8 ± 1.1 MPa, 86.0 ± 0.9 MPa, 88.9 ± 1.2 MPa, and 69.9 ± 0.9 MPa respectively. Ultrasonic pulse velocity testing and strength degradation calculation results as well as visual analysis of specimens during thermal cycling (Figure 4) suggest that a small amount of HCM in refractory castable can reduce the formation and propagation of cracks and thus increase its thermal shock resistance. 

The modulus of elasticity calculated from the Equation (2) for specimens K0, K2.5, K5, and K10 subjected to firing at 950 °C was 35.4 ± 0.2 GPa, 30.2 ± 0.3 GPa, 33.0 ± 0.7 GPa, and 33.9 ± 0.1 GPa, respectively. The results of the present work correlate with the data reported in the literature. Researchers [28] claim that thermal shock resistance of castables improves by lowering the modulus of elasticity. 

Stress-to-elastic modulus ratio (σ/E), characterizes the ability of a material to resist crack nucleation, and strength damage factor, showing theoretical drop in material compressive strength after thermal cycling for K0, K2.5, K5, and K10 reach 3.2 ± 0.10, 3.6 ± 0.08, 3.5 ± 0.09, and 2.9 ± 0.06 respectively. These results show, that a stronger material (compositions K0, K2.5, and K5, Figure 3) with a lower modulus of elasticity (especially K2.5) have a higher thermal resistance.

**Figure 6:**
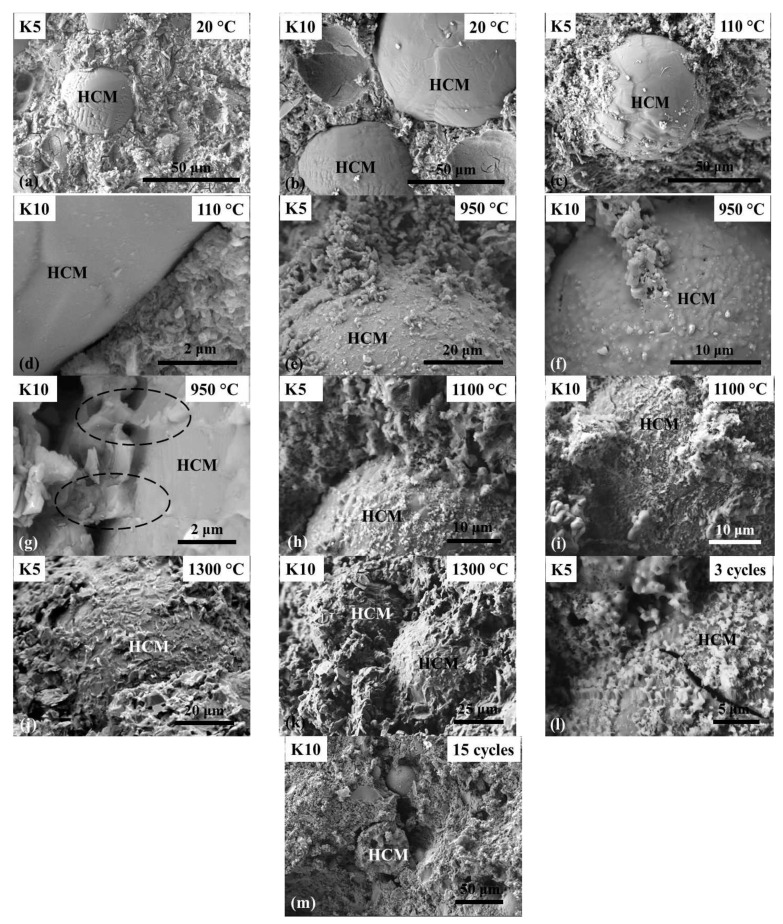
SEM images of the structure of castables containing 5% and 10% of HCM after hardening at 20 °C (**a**,**b**), drying at 110 °C (**c**,**d**), firing at 950 °C (**e**–**g**), 1100 °C (**h**,**i**), and 1300 °C (**j**,**k**), and after 3 and 15 thermal cycles (**l**,**m**).

The microstructure of castables was investigated by SEM. The HCM distribution in the structure of castable and the contact zone between the HCM and the binder were analyzed (Figure 6). A fairly uniform distribution of particles was observed in the structure of castable specimens containing up to 5% of HCM with each microsphere surrounded by the binder layer, tightly adjoining the particle surface (Figure 6a). As HCM are inert, they do not react with binder. That is evidenced by the clean, smooth, unchanged surface of HCM particles after hardening (Figure 6a,b) and drying of castable at 110 °C (Figure 6c,d), as well as the well-defined interface line between HCM surface and the binder (Figure 6d). The firing of castable at 950 °C resulted in an altered morphology of the particle surface (Figure 6e,f) and the formation of common phases in the HCM-binder contact zone (Figure 6g), which, in turn, ensure their bonding. A further change in HCM surface morphology and increased area of HCM adhesion with the binder (Figure 6h,i) was observed at the higher firing temperature of 1100 °C. At 1300 °C, the sintering of HCM with the matrix occurred all over the contact area (Figure 6j,k). After three thermal cycles at 950 °C, micro-cracks were detected on the surface of some HCM particles (Figure 6l). It can be assumed that the formation of cracks and fracture of HCM (presumably the thin-walled microspheres) occurs under the influence of compressive stresses caused by thermal deformation of castable while heating. Since part of the energy is spent on the fracturing of HCM, the overall stress level is reduced and thus significantly fewer micro-cracks are formed in hardened cement paste. It can also be assumed that the regular spherical shape of HCM particles has a positive effect. Stress concentration commonly occurs in the point of structure defects. A regular spherical shape has the lowest stress concentration factor compared to other shapes. Thus, evenly distributed in the matrix microspheres act not only as traps for cracks inhibiting crack propagation, but also reduce the level of stress concentration and more effectively suppress the propagation of microcracks at the binder and HCM interface. 

A higher occurrence of microsphere clusters was observed in castables containing a higher amount of HCM (10%). In this case, the matrix does not completely cover the surface of the particle and spaces remain between the particles or a thin layer of matrix is formed between the particles, which breaks down when concrete is deformed during thermal loads, subsequently, forming cavities (Figure 6b). Thus, coarser void of irregular shape instead of regular spherical void is formed in the matrix in the location of microspheres cluster, diminishing the positive effect from HCM addition in such a way. It was determined also that during thermal cycling, macro-cracks passed through HCM clusters and joined the voids formed in the structure (Figure 6m). As is known, the presence of large, irregularly distributed voids across the material volume impairs the mechanical properties of castable. This impairment is illustrated by decreased CS and reduced thermal shock resistance of castable containing 10% of HCM.

## 4. Conclusions

The effect of hollow corundum microspheres (HCM) on both physical mechanical properties and thermal shock resistance behavior of refractory medium cement castable with bauxite aggregate was studied in the present work. The water quench test and nondestructive ultrasonic testing method along with calculated strength degradation and σ/E ratio were used to evaluate and compare thermal shock resistance of refractory castable. The following conclusions were drawn from the results of experimental research described in this paper:

A 2.5% and 5% addition of 0–100 μm size HCM improves the thermal resistance of alumina cement-based refractory castable without significantly affecting its density and compressive strength: as compared with reference samples, for these specimen series, the appearance of fewer outside cracks was established by visual control during water quench testing up to 30 cycles while less drop in ultrasonic pulse velocity revealed fewer inside damages. Stress-to-elastic modulus ratio (σ/E), characterizing the ability of a material to resist crack nucleation, and strength degradation, showing theoretical drop in material compressive strength after thermal cycling, were improved by approximately 12% and 10%, respectively. The most probable mechanism for increasing thermal resistance is the reduction of stress concentration in the material due to the regular spherical shape of the HCM. 

The deterioration of thermal shock resistance parameters and compressive strength was observed with the increase of HCM mass fraction in castable up to 10%: the appearance of the outside cracks was comparable with that of reference samples while other parameters (Vup, σ/E, and strength degradation) were even worse. Such worsening effect may be associated with the clustering of HCM particles in the matrix, which was observed during microstructural analysis and, in terms of material matrix continuity, can be considered as a formation of coarser non-uniformly distributed cavities of irregular shape, that may cause the increase in stress concentration level.

## Figures and Tables

**Table 1:** Composition of castables.

Grade	G70, g	MC, g	RA, g	CA, g	Bauxite 0–0.1 mm, g	Bauxite 1–3 mm, g	Bauxite 0–1 mm, g	PCE, g *	TP, g *	HCM, g *	Water, g *
K0	1440	360	600	600	1200	5400	2400	12	12	0	840
K2.5	1440	360	600	600	900	5400	2400	12	12	300	840
K5	1440	360	600	600	600	5400	2400	12	12	600	840
K10	1440	360	600	600	0	5400	2400	12	12	1200	840

*—above 100%, calculated according mass of dry materials.

## Data Availability

Not applicable.
